# Impact of hormonal crosstalk on plant resistance and fitness under multi-attacker conditions

**DOI:** 10.3389/fpls.2015.00639

**Published:** 2015-08-17

**Authors:** Irene A. Vos, Liselotte Moritz, Corné M. J. Pieterse, Saskia C. M. Van Wees

**Affiliations:** Plant-Microbe Interactions, Department of Biology, Faculty of Science, Utrecht UniversityUtrecht, Netherlands

**Keywords:** hormonal crosstalk, resistance, fitness, *Hyaloperonospora arabidopsidis*, *Pieris rapae*, *Botrytis cinerea*

## Abstract

The hormone salicylic acid (SA) generally induces plant defenses against biotrophic pathogens. Jasmonic acid (JA) and its oxylipin derivatives together with ethylene (ET) are generally important hormonal regulators of induced plant defenses against necrotrophic pathogens, whereas JAs together with abscisic acid (ABA) are implicated in induced plant defenses against herbivorous insects. Hormonal crosstalk between the different plant defense pathways has often been hypothesized to be a cost-saving strategy that has evolved as a means of the plant to reduce allocation costs by repression of unnecessary defenses, thereby minimizing trade-offs between plant defense and growth. However, proof for this hypothesis has not been demonstrated yet. In this study the impact of hormonal crosstalk on disease resistance and fitness of *Arabidopsis thaliana* when under multi-species attack was investigated. Induction of SA- or JA/ABA-dependent defense responses by the biotrophic pathogen *Hyaloperonospora arabidopsidis* or the herbivorous insect *Pieris rapae*, respectively, was shown to reduce the level of induced JA/ET-dependent defense against subsequent infection with the necrotrophic pathogen *Botrytis cinerea*. However, despite the enhanced susceptibility to this second attacker, no additional long-term negative effects were observed on plant fitness when plants had been challenged by multiple attackers. Similarly, when plants were grown in dense competition stands to enlarge fitness effects of induced defenses, treatment with a combination of SA and MeJA did not cause additional negative effects on plant fitness in comparison to the single MeJA treatment. Together, these data support the notion that hormonal crosstalk in plants during multi-attacker interactions allows plants to prioritize their defenses, while limiting the fitness costs associated with induction of defenses.

## Introduction

Plants can activate defense responses to protect themselves against a plethora of microbial pathogens and herbivorous insects. These defense responses are modulated by the induced production of a hormonal blend in the plant. The plant hormones salicylic acid (SA), jasmonic acid (JA), ethylene (ET), and abscisic acid (ABA) are important regulators of induced defense mechanisms (Robert-Seilaniantz et al., 2011; Pieterse et al., 2012; Vos et al., 2013a). SA-dependent defenses are generally effective against biotrophic pathogens, while JA-dependent defenses are generally effective against necrotrophic pathogens and herbivorous insects (Glazebrook, 2005; Howe and Jander, 2008). SA is rapidly synthesized upon infection with biotrophic pathogens (Malamy et al., 1990; Métraux et al., 1990). Defense signaling downstream of SA depends on the transcriptional regulator NPR1 (Dong, 2004), eventually resulting in the activation of a large set of defense-related genes, amongst which the robust marker gene of the SA signaling pathway, *PR1* (Van Loon et al., 2006). In response to wounding, insect herbivory or infection with necrotrophic pathogens, JA and its oxylipin derivatives (collectively referred to as jasmonates) rapidly accumulate in plants (Creelman et al., 1992; Penninckx et al., 1996). In *Arabidopsis thaliana* (*Arabidopsis*), there are two distinct branches of the JA response pathway that antagonize each other; the ERF-branch and the MYC-branch (hereafter referred to as such). The ERF-branch is activated upon infection with necrotrophic pathogens and is regulated by the AP2/ERF-domain transcription factors ERF1 and ORA59 (Anderson et al., 2004; Pré et al., 2008). The ERF-branch of the JA response is co-regulated by ET and results in activation of a large set of ERF-branch genes, including the marker gene *PDF1.2* (Penninckx et al., 1998; Lorenzo et al., 2003). The MYC-branch is activated upon wounding or feeding by herbivorous insects and is regulated by the basic helix-loop-helix leucine zipper transcription factors MYC2, MYC3, and MYC4 in concerted action with ABA (Anderson et al., 2004; Fernández-Calvo et al., 2011; Niu et al., 2011; Vos et al., 2013b). Activation of the MYC-branch leads to transcription of a large set of JA-responsive genes, including *VSP1* and *VSP2* that are marker genes of the MYC-branch (Anderson et al., 2004; Lorenzo et al., 2004).

Activation of the different hormone-regulated defense responses is not without fitness costs. In several plant species it has been shown that exogenous application of SA or its chemical analog benzothiadiazole (BTH) inhibited plant growth and seed production (Heil et al., 2000; Cipollini, 2002; Canet et al., 2010). Furthermore, under non-infected conditions, *Arabidopsis* mutants constitutively expressing SA-dependent defenses are dwarfed and severely affected in seed production (Bowling et al., 1994; Heil and Baldwin, 2002; Heidel et al., 2004; Van Hulten et al., 2006). Conversely, SA-deficient *Arabidopsis* genotypes have higher growth rates and seed production compared to wild-type plants (Cipollini, 2002; Abreu and Munné-Bosch, 2009). Activation of JA-dependent defense responses can also result in negative effects on plant fitness. Infestation with insects or exogenous application of JA decreased seed production and delayed flowering and fruit ripening (Agrawal et al., 1999; Redman et al., 2001; Van Dam and Baldwin, 2001). In addition, *Arabidopsis* plants constitutively expressing JA-dependent defenses, showed reduced growth phenotypes (Ellis and Turner, 2001; Cipollini, 2010). Together, this demonstrates the negative fitness effects of SA- and JA-mediated defense activation. Other hormones like brassinosteroids, gibberellins, and auxin have recently emerged as crucial regulators of the defense-growth trade-off induced by pathogens (Denancé et al., 2013; Lozano-Durán and Zipfel, 2015). The growth repression induced by SA and JA is most likely also mediated via signal integration with the growth hormones (Huot et al., 2014).

Quantity, composition and timing of the hormonal blend and cross-communication between the hormone signaling pathways contributes to activation of effective over infective defenses (De Vos et al., 2005; Pieterse et al., 2012; Vos et al., 2013a; Caarls et al., 2015). Many cases of crosstalk between the SA and JA defense pathway have been reported (Bostock, 2005; Stout et al., 2006; Pieterse et al., 2012). Pharmacological experiments with *Arabidopsis* revealed that the JA-responsive genes *PDF1.2* and *VSP2* are highly sensitive to suppression by SA. The antagonistic effect of SA on JA signaling was observed in a large number of *Arabidopsis* accessions (Koornneef et al., 2008) and was even reported to remain active in the next generation of plants (Luna et al., 2012), highlighting the potential significance of this phenomenon in the regulation of induced plant defenses in nature. This antagonism between SA and JA signaling can affect plant resistance. For example, in *Arabidopsis*, induction of the SA pathway by exogenous application of SA or infection with the hemibiotrophic pathogen *Pseudomonas syringae*, rendered the plants more susceptible to the necrotrophic fungus *Alternaria brassicicola* (Spoel et al., 2007; Leon-Reyes et al., 2009). Furthermore, reduced SA signaling in *Arabidopsis* genotypes *NahG* and *npr1* was correlated with reduced feeding by the herbivorous insect *Trichoplusia ni* (Cui et al., 2002).

Likewise, between the ERF- and the MYC-branch of the JA pathway a mutually antagonistic relationship exists (Lorenzo et al., 2004; Verhage et al., 2011; Vos et al., 2013b). This antagonism between the ERF- and the MYC-branch can affect plant resistance against necrotrophs. For example, in *MYC2*-mutated *jin1* and ABA biosynthesis mutant *aba2-1* plants, the ERF-branch of the JA pathway is stimulated, resulting in enhanced resistance against necrotrophic pathogens, such as *Botrytis cinerea, Plectosphaerella cucumerina*, and *Fusarium oxysporum* (Anderson et al., 2004; Lorenzo et al., 2004; Nickstadt et al., 2004; Adie et al., 2007; Sánchez-Vallet et al., 2012). Furthermore, caterpillars of the insect herbivore *Pieris rapae* preferred to feed from *jin1* mutant plants and *ORA59*-overexpressing plants over wild-type plants (Verhage et al., 2011), indicating that crosstalk between the ERF- and the MYC-branch also affects plant–insect interactions.

Extensive cross-communication between defense signaling pathways allows the plant to fine-tune the defense response to the attacker at hand (Reymond and Farmer, 1998). Since activation of inducible plant defenses is not without costs, there are trade-offs between plant defense and growth (Heil and Baldwin, 2002; Van Hulten et al., 2006; Walters and Heil, 2007; Vos et al., 2013a; Cipollini et al., 2014). Hormonal crosstalk has often been interpreted as a cost-saving strategy and may have evolved as a means of the plant to reduce allocation costs by repression of unnecessary defenses that are ineffective against the attacker that is encountered (Pieterse and Dicke, 2007; Thaler et al., 2012).

In this study the impact of hormonal crosstalk on disease resistance and fitness of *Arabidopsis* plants when under multi-species attack was investigated. Induction of SA- or JA/ABA-dependent signaling induced by a primary attacker was shown to negatively affect JA/ET-dependent defense responses activated by subsequent attack with a necrotrophic pathogen, resulting in reduced resistance to this attacker. However, although plants under multi-species attack became more susceptible to the second attacker, this did not lead to long-term negative fitness effect, providing preliminary support for the cost-saving character of hormonal crosstalk.

## Results

### Multi-Attacker Conditions Reduce Resistance but not Fitness of *Arabidopsis* Plants

**FIGURE 1:**
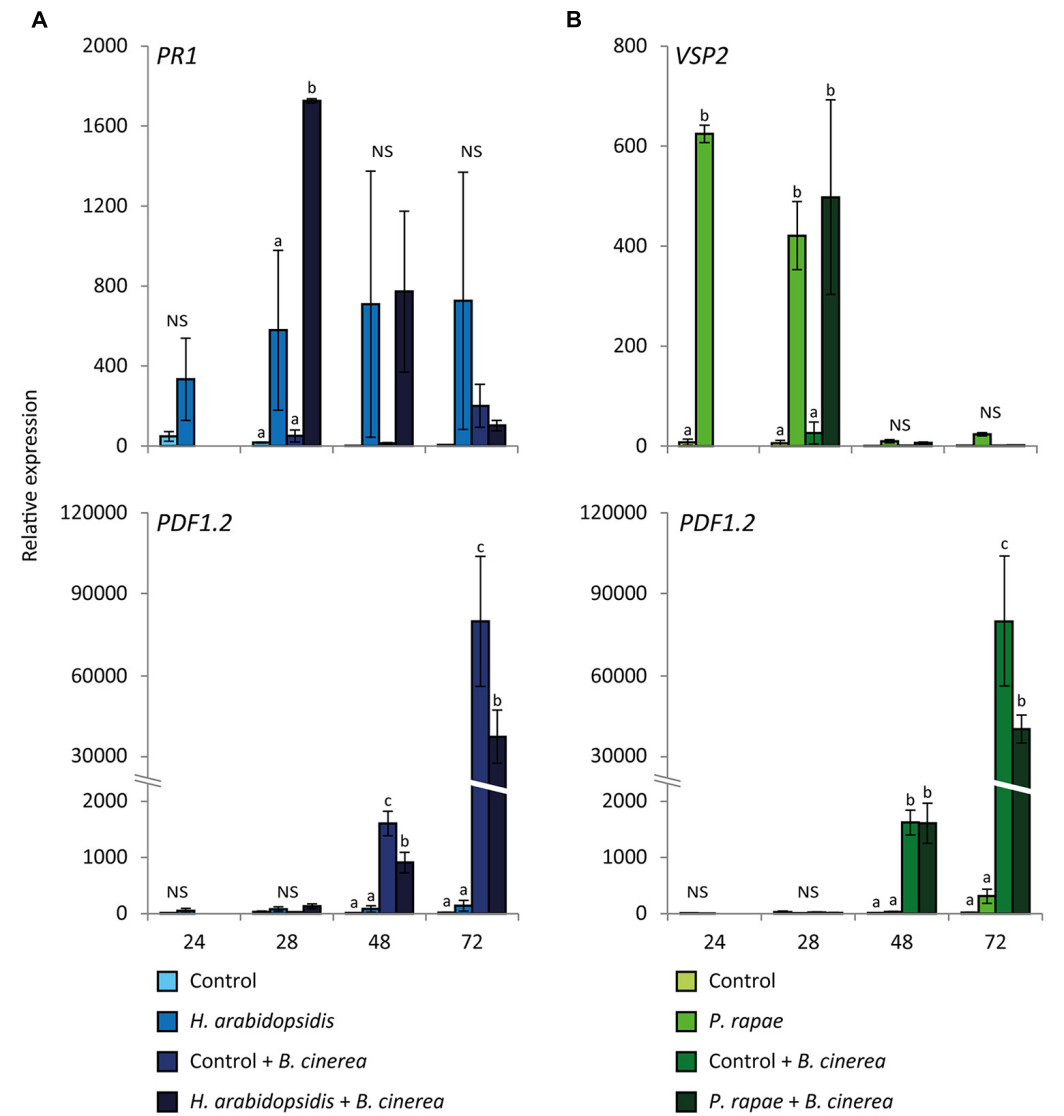
**Differential expression of *PR1, VSP2*, and *PDF1.2* in response to multiple attackers.** RT-qPCR analysis of *Hyaloperonospora arabidopsidis*-responsive *PR1* expression **(A)**, *Pieris rapae*-responsive *VSP2* expression **(B)** and *Botrytis cinerea*-responsive *PDF1.2* expression **(A,B)**. Plants were either inoculated with *H. arabidopsidis* or infested with *P. rapae* caterpillars. At 24 h the caterpillars were removed after which all plants were inoculated with *B. cinerea*. Samples were taken at the indicated time points after the first treatment. Different letters indicate a statistically significant difference between the different treatments within one time point (ANOVA, Tukey *post hoc* test; *P* < 0.05; NS, not significant). Error bars represent SE, *n* = 3 plants.

In this research, fitness costs associated with defense against multiple attackers were investigated. To this end, 5-week-old *Arabidopsis* plants were exposed to two attackers that induce antagonizing defense pathways. Firstly, the plants were either inoculated with the biotrophic pathogen *Hyaloperonospora arabidopsidis*, which induces the SA pathway, or infested with *P. rapae* caterpillars, which induce the MYC-branch of the JA pathway. Twenty-four hour later, the caterpillars were removed after which all plants were inoculated with the necrotrophic pathogen *B. cinerea*, which induces the ERF-branch of the JA pathway. **Figure 1** shows the gene expression results from the defense inductions by the different combinations of attackers. When plants were infected with *H. arabidopsidis*, expression of the SA pathway marker gene *PR1* was enhanced, although the induction was not statistically significant due to high variation between the biological replicates (**Figure 1A**). In the combination treatment of *H. arabidopsidis* and *B. cinerea, PR1* was significantly induced at 28 h, probably because *B. cinerea* triggers the SA pathway as a virulence strategy (El-Oirdi et al., 2011) and the tissue may be primed for SA responsiveness by the *H. arabidopsidis* infection. *PR1* expression leveled off again toward 72 h. Feeding by *P. rapae* induced the MYC-branch, as indicated by high *VSP2* expression (**Figure 1B**). *VSP2* expression returned to basal levels at 48 h and was not altered in the combination treatment with *B. cinerea* at any of the time points investigated. In all cases, the ERF-branch marker gene *PDF1.2* was activated in response to *B. cinerea* infection at 48 and 72 h, but was strongly repressed when plants were previously infected with *H. arabidopsidis* or infested with *P. rapae* (**Figures 1A,B**). Similar antagonistic effects on *PDF1.2* gene expression were found when, before *B. cinerea* infection, plants were induced by exogenous application of either 1 mM SA or a combination of 100 μM MeJA and 100 μM ABA (**Supplementary Figure S1**). This indicates that the activation of the SA pathway or the MYC-branch of the JA pathway prior to infection with *B. cinerea* suppressed the *B. cinerea*-induced activation of the ERF-branch, providing evidence for hormonal crosstalk on defense gene expression level induced by combinations of different attackers.

**FIGURE 2:**
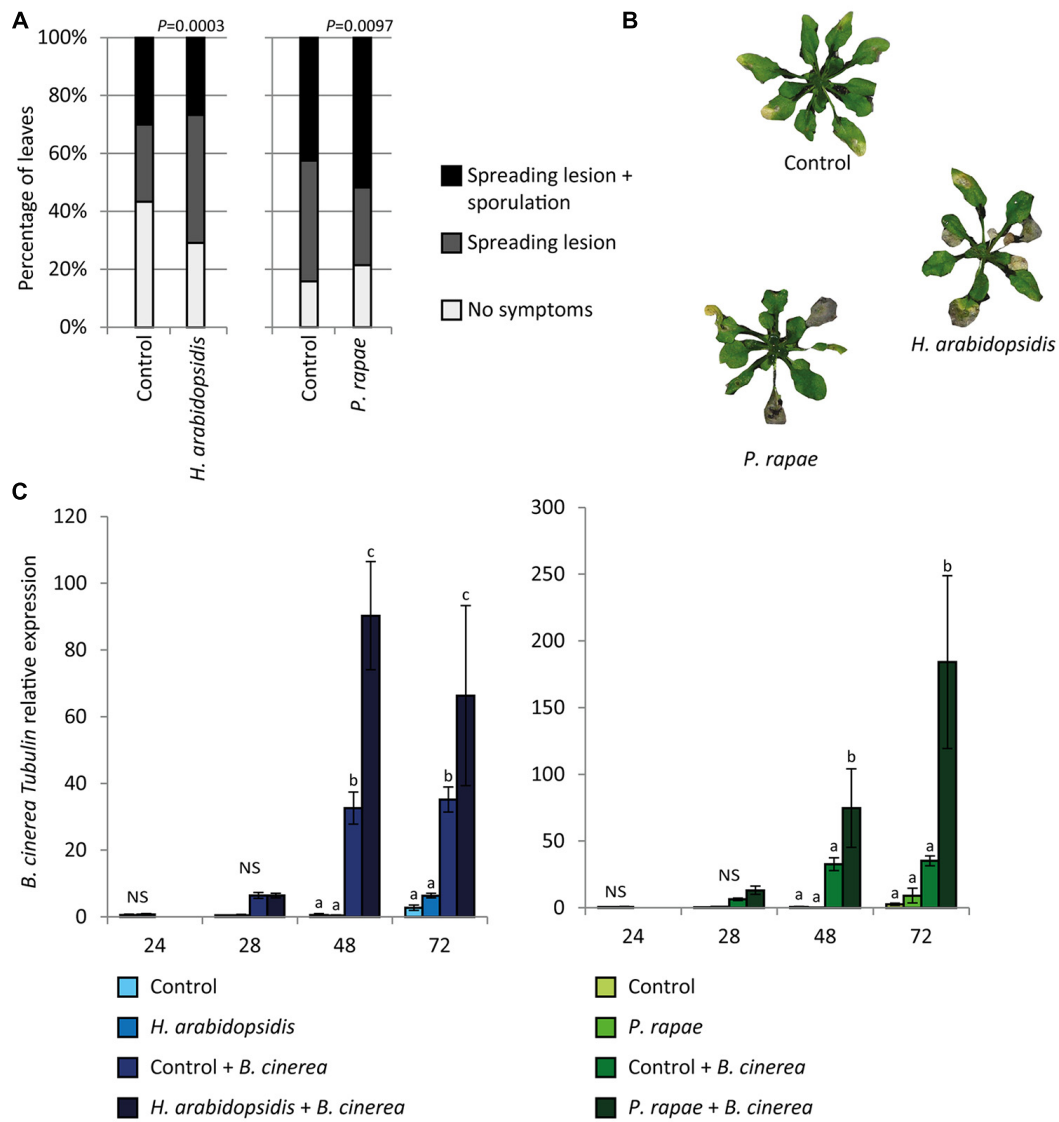
**Effect of prior attack by *H. arabidopsidis* or *P. rapae* on disease resistance against *B. cinerea*. (A)** Quantification of disease symptoms of *Arabidopsis* Col-0 plants infected with *B. cinerea*. Twenty-four hour before inoculation with *B. cinerea*, plants were inoculated with *H. arabidopsidis* or infested with *P. rapae*. Disease severity of the inoculated leaves was scored in three classes. Percentage of leaves in each class was calculated per plant (*X*^2^-test; *n* = 20 plants). **(B)** Disease symptoms of *B. cinerea* infection in control plants, *H. arabidopsidis*-induced plants and *P. rapae*-induced plants. **(C)** RT-qPCR analysis of *B. cinerea Tubulin* levels relative to *Arabidopsis* reference gene mRNA levels after single and double treatments. Samples were taken at the indicated time points after the first treatment **(A,B)**. Different letters indicate a statistically significant difference between the different treatments within one time point (ANOVA, Tukey *post hoc* test; *P* < 0.05; NS, not significant). Error bars represent SE, *n* = 3 plants.

To investigate whether suppression of the ERF-branch by prior attack with either *H. arabidopsidis* or *P. rapae* is accompanied by a reduced level of resistance against *B. cinerea*, we performed disease resistance bioassays. Plants that were induced by *H. arabidopsidis* or *P. rapae* were significantly more susceptible to *B. cinerea* than control plants (**Figures 2A,B**). Accordingly, *B. cinerea Tubulin* transcript levels were significantly higher in induced plants than in control plants (**Figure 2C**). Plants that were treated with exogenous application of 1 mM SA or a combination of 100 μM MeJA and 100 μM ABA were also more susceptible to subsequent *B. cinerea* infection (**Supplementary Figure S2**). Together, these results show that suppression of the ERF-branch of the JA pathway by either the SA inducer *H. arabidopsidis* or the MYC-branch inducer *P. rapae* coincides with a reduction in the level of resistance against *B. cinerea*.

**FIGURE 3:**
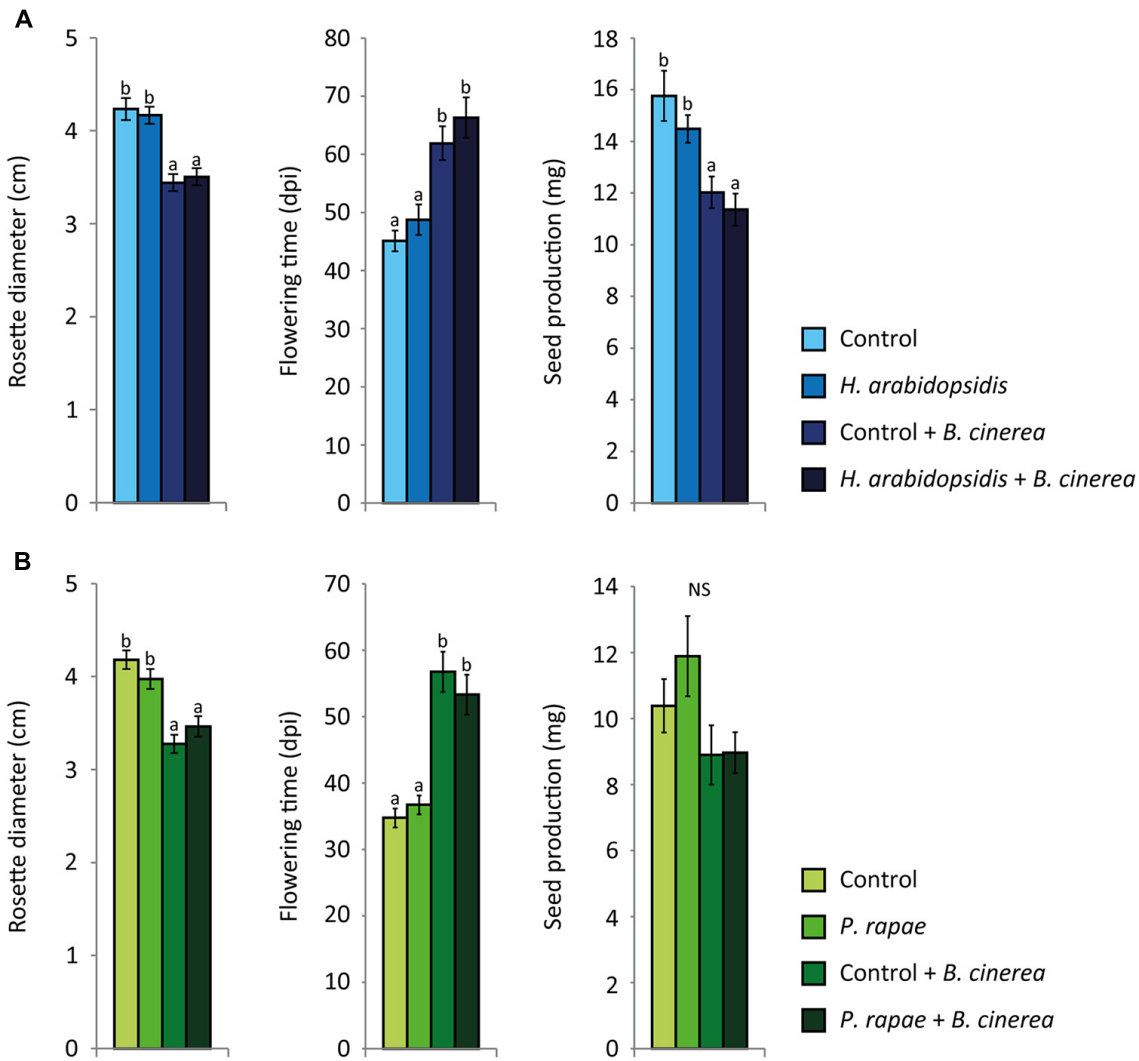
**Growth and fitness parameters of single- and double-attacked plants.** Rosette diameter (cm), flowering time (days post inoculation), and total seed production (mg) of *Arabidopsis* plants. Plants were either inoculated with *H. arabidopsidis* **(A)** or infested with *P. rapae* caterpillars **(B)**. At 24 h the caterpillars were removed after which all plants were inoculated with *B. cinerea* **(A,B)**. Different letters indicate a statistically significant difference between the different treatments (ANOVA, Tukey *post hoc* test; *P* < 0.05; NS = not significant). Error bars represent SE, *n* = 20 plants.

To investigate whether these hormonal crosstalk-mediated effects on *PDF1.2* gene expression and resistance to *B. cinerea* impacted the fitness of the plants under multi-attacker conditions, the rosette size, flowering time and seed production were measured. Neither *H. arabidopsidis* infection nor *P. rapae* infestation affected any of these fitness parameters by themselves (**Figures 3A,B**), which could be explained by the non-optimal temperature for infection with *H. arabidopsidis* from 24 h onward and the removal of the *P. rapae* caterpillars at 24 h. In contrast, *B. cinerea* infection had a strong negative effect on rosette size and seed production and prolonged the flowering time (**Figures 3A,B**). Prior attack with either *H. arabidopsidis* or *P. rapae* did not result in an additional effect on the fitness traits compared to *B. cinerea* infection alone. Similar results were found when plants were induced by exogenous application of 1 mM SA or a combination of 100 μM MeJA and 100 μM ABA (**Supplementary Figure S3**). Overall, it can be concluded that infection with *B. cinerea* led to reduced fitness. Nonetheless, although prior attack by *H. arabidopsidis* or *P. rapae* or induction by exogenously applied SA or a combination of MeJA and ABA resulted in enhanced susceptibility to *B. cinerea* infection, which was likely due to the suppression of the ERF-branch, this was not associated with additional fitness costs.

### Fitness Costs of SA and MeJA Treatments in Competition-Grown Plants

**FIGURE 4:**
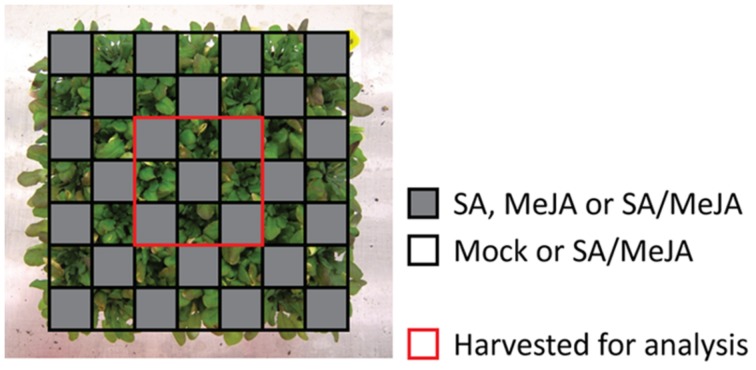
**Schematic overview of the competition experiment set-up.** *Arabidopsis* plants were grown in competition trays, consisting of separate small pots positioned very close together. Each tray consisted of 49 plants, of which 25 plants were soil-drenched containing 500 μM SA, 50 μM MeJA, or a combination of both hormones. The other 24 plants were treated with either a mock solution or a combination of both hormones. Only the inner nine plants were used for measurements, to circumvent any edge effect.

**FIGURE 5:**
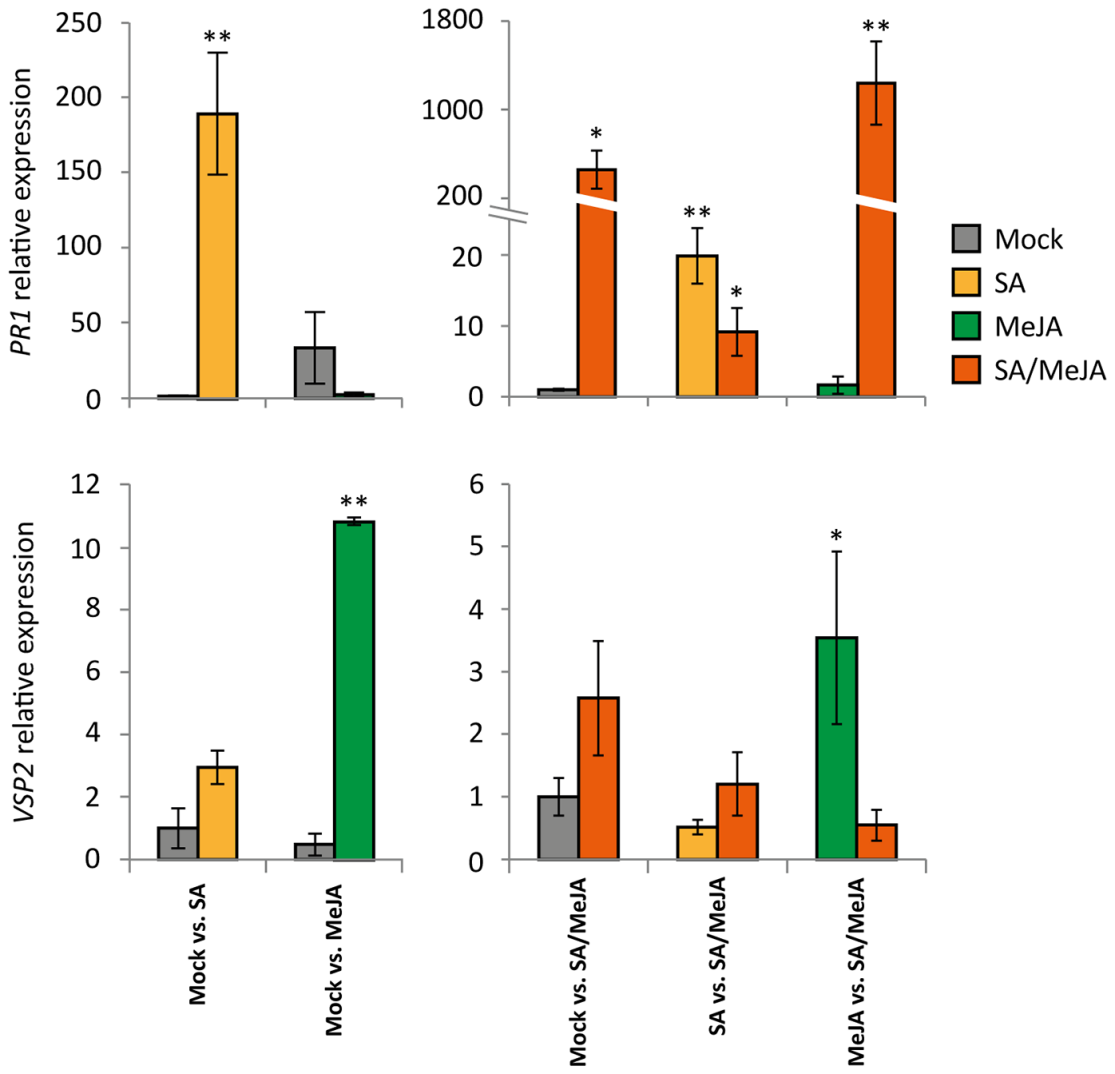
**Differential gene expression in competition-grown plants.** RT-qPCR analysis of salicylic acid (SA)-responsive *PR1* expression and Jasmonic acid (JA)-responsive *VSP2* expression in competition-grown plants 24 h after treatment with a mock, SA, MeJA, or SA/MeJA solution. Mock vs. SA and Mock vs. MeJA competition experiments were performed in one experimental round, and Mock vs. SA/MeJA, SA vs. SA/MeJA, and MeJA vs. SA/MeJA competition experiments were performed in another experimental round. Indicated are expression levels relative to those of mock-treated plants in one of the competition trays. Asterisks indicate a statistically significant difference between the indicated treatment and the mock-treated plants (ANOVA, Dunnet *post hoc* test; ^∗∗^ = *P* < 0.01; ^∗^ = *P* < 0.05). Error bars represent SE, *n* = 3 plants.

Since competition for light and nutrients can increase the probability of detecting fitness costs of activating different hormone signaling pathways (Dietrich et al., 2005), *Arabidopsis* plants were grown in competition trays, consisting of separate small pots positioned very close together. This set-up led to competition of the above-ground plant parts, but not of the root-systems. Each tray consisted of 49 plants, of which 25 plants were supplied with a soil drench containing 500 μM SA, 50 μM MeJA, or a combination of both hormones. The other 24 plants were treated with either a mock solution or a combination of both hormones (**Figure 4**). Only the inner nine plants were used for measurements, to circumvent any edge effect. In all trays, SA and SA/MeJA treatment induced *PR1* expression, whereas *VSP2* expression was only induced by the single MeJA treatment and not by the SA/MeJA combination treatment (**Figure 5**), confirming that the hormone treatments induced the expected effects on SA- and JA-responsive gene expression.

**FIGURE 6:**
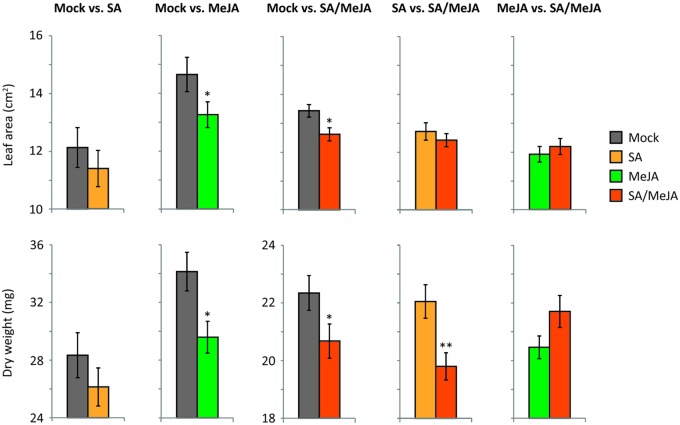
**Growth parameters in competition-grown plants.** Leaf area (cm^2^) and dry weight (mg) of the rosettes of competition-grown plants 3 weeks after treatment with a mock, SA, MeJA, or SA/MeJA solution. Mock vs. SA and Mock vs. MeJA competition experiments were performed in one experimental round, and Mock vs. SA/MeJA, SA vs. SA/MeJA and MeJA vs. SA/MeJA competition experiments were performed in another experimental round. Asterisks indicate a statistically significant difference between the two treatments of the indicated competition tray (Students *t*-test; ^∗∗^*P* < 0.01; ^∗^*P* < 0.05). Error bars represent SE, *n* = 20–25 plants.

When MeJA- or SA/MeJA-treated plants competed with mock-treated plants, leaf area and dry weight of the hormone-treated plants were reduced compared to the mock-treated plants (**Figure 6**). SA-treated plants did not show a significant reduction in leaf area or dry weight in competition with mock-treated plants, although a trend toward a reduction in leaf area and dry weight was detected. There was no significant difference in leaf area or dry weight when MeJA- and SA/MeJA-treated plants competed with each other, although a trend toward increased dry weight and leaf area was observed in the double treatment. Together, this indicates that there was no extra negative fitness effect of the double treatment compared to the MeJA treatment alone, but rather a trend toward a reduction of MeJA-induced fitness costs in the double treatment. On the other hand, when SA-treated plants competed with SA/MeJA-treated plants, SA/MeJA-treated plants had lower dry weight than SA-treated plants, but there was no significant difference in leaf area in this competition. The observed differences in *PR1* and *VSP2* expression levels and in growth between plants that had received the same treatment but were placed in different competition trays can likely be ascribed to their dependency on the competition partner, but possibly also to unexpected environmental differences between trays or between experimental rounds. Still, the within tray comparisons show that especially the activation of the JA pathway resulted in lower fitness and lower competitive ability. Activation of the SA pathway did not have major negative effects on fitness in a competitive environment. Treatment with a combination of SA and MeJA reduced plant fitness, but did not result in an extra negative effect compared to the single MeJA treatment, indicating that also in dense competition stands, hormonal crosstalk might be a cost-saving strategy in induced plant immunity.

## Discussion

Crosstalk between hormone-regulated defense pathways is suggested to allow plants to fine-tune their defenses to optimize induced resistance to an attacker and reduce allocation costs (Heil and Baldwin, 2002; Pieterse and Dicke, 2007; Walters and Heil, 2007; Vos et al., 2013a). Therefore, hormonal crosstalk has often been suggested to be a cost-saving strategy. Since individual plants are likely to be attacked by more than one organism, hormonal crosstalk may coincide with ecological costs, leading to higher susceptibility to a subsequent attacker (Heil, 2002; Vos et al., 2013a). Moreover, not much is known about the consequences of hormonal crosstalk on plant fitness under multi-species attack (Thaler et al., 2012). In this research, costs of defense activation of *Arabidopsis* plants by multiple attackers or hormones were investigated.

### Costs and Benefits of *Arabidopsis* Plants Under Multi-Species Attack

Several studies found a negative effect on resistance against a subsequent attacker when a plant was previously induced by another attacker (Vos et al., 2013a). For example, in *Arabidopsis*, infection with the hemibiotrophic pathogen *P. syringae* resulted in higher susceptibility to a subsequent infection with the necrotrophic fungus *A. brassicicola* (Spoel et al., 2007). Furthermore, feeding by the generalist herbivore *Spodoptera littoralis* led to increased growth of a virulent strain of *P. syringae* (Appel et al., 2014). In tobacco, *Manduca sexta* caterpillars consumed up to 2.5-times more leaf tissue from plants previously infected with the SA-inducing tobacco mosaic virus than from mock-treated plants (Preston et al., 1999) and black bean aphids had a higher growth rate and fecundity on bean leaves infected with the necrotrophic pathogen *Botrytis fabae*, compared to uninfected leaves (Zebitz and Kehlenbeck, 1991). However, very few studies have investigated whether and how attack by multiple attackers affect plant fitness (Hauser et al., 2013). In this study we found that when *Arabidopsis* plants were first induced by the SA pathway-inducing pathogen *H. arabidopsidis* or the MYC-branch-inducing caterpillar *P. rapae*, the ERF-branch of the JA pathway was suppressed and plants became more susceptible to a subsequent attack by the necrotrophic pathogen *B. cinerea* compared to non-induced plants (**Figures 1** and **2**). Likewise, pretreatment with the hormones SA or a combination of MeJA and ABA, suppressed the induction of the ERF-branch and resulted in higher susceptibility to *B. cinerea* (**Supplementary Figures S1** and **S2**).

Infection with *B. cinerea* or exogenous application of MeJA activated defense gene expression (**Figures 1** and **5**). Furthermore, a negative effect on growth, flowering time and seed production was found after infection with *B. cinerea* (**Figure 3**) or MeJA treatment (**Figure 6**), suggesting that there were trade-offs between activation of defenses by these treatments and plant fitness (Heil and Baldwin, 2002; Van Hulten et al., 2006; Walters and Heil, 2007; Vos et al., 2013a; Cipollini et al., 2014). However, there was no negative effect on growth, flowering time and seed production in response to *H. arabidopsidis* infection or infestation with *P. rapae*, while these attackers also induced defense gene expression (**Figures 1** and **3**). This is probably caused by the fact that plants were only shortly exposed to these attackers, since 24 h after the first pathogen or insect treatment, plants were placed under conditions that inhibited further growth of the pathogen (*H. arabidopsidis*) or the inducer was removed (*P. rapae*), thereby reducing long-term effects of the primary induction treatments on plant fitness. Although these single inductions did not have a negative effect on the fitness parameters, still there were ecological costs of the double induction, as plants became more susceptible to *B. cinerea* infection (**Figure 2**), which provides us with a great power to actually study the effect of crosstalk on plant fitness. These ecological costs did not lead to additional negative effects on fitness compared to *B. cinerea*-infected plants that were not previously induced (**Figure 3**). Likewise, no additional fitness costs were incurred by the double treatment with SA or a combination of MeJA and ABA and *B. cinerea* (**Supplementary Figure S3**). Together, this could be an indication that the hormonal crosstalk effect at the level of gene expression (**Figure 1**; **Supplementary Figure S1**) and disease resistance (**Figure 2**; **Supplementary Figure S2**) is indeed a cost-saving strategy. Alternatively, there might not be a linear relation between susceptibility and plant fitness, which could explain the lack of additional fitness costs in the double treated plants. However, Heidel et al. (2004) found that higher disease severity after *H. arabidopsidis* infection correlated with lower seed production. Furthermore, Cipollini (2002) found that seed production was significantly lower when plants were treated with a high concentration of SA compared to a lower concentration. Together this suggests that there is a negative correlation between disease symptoms and fitness. To our knowledge this has never been shown for *B. cinerea* infection, but our own experience is that *Arabidopsis* can die from a *B. cinerea* infection, leading to a fitness level of 0.

Similar to our results, Van Mölken et al. (2014) found that flea beetle larvae caused more damage on *Barbarea vulgaris* plants that were infected with the biotrophic oomycete *Albugo* sp., but this did not lead to a difference in seed production. However, a meta-analysis study by Hauser et al. (2013) indicated that general conclusions on interactive effects by multiple attackers on plant fitness cannot be drawn. They showed that most of the measured plant fitness traits in different studies could be only weakly explained by interactive effects of herbivores and pathogens in general, although environmental conditions and overall infection load could in some cases lead to synergistic or antagonistic fitness impacts by the combination treatments. Testing crosstalk mutants that are not affected in resistance to either of the attackers could give a clearer answer to the question whether hormonal crosstalk is indeed a cost-saving strategy (Thaler et al., 2012; Vos et al., 2013a).

### Fitness Effects of Defense Activation in Plants Grown in Competition

Previously, it was shown in *Nicotiana attenuate* plants that allocation costs of induced defenses were only found when plants were grown with conspecific competitors (Van Dam and Baldwin, 2001). This can likely be explained by the fact that in dense competition stands, plants have to compete for light and nutrients in addition to the investment of resources in activation of induced defenses. Consequently, growing plants in a competition set-up can increase the probability of detecting fitness costs of activating the different defense signaling pathways (Dietrich et al., 2005). Therefore, we tested the effect of hormone treatments on the fitness of plants grown in dense competition stands.

Salicylic acid and/or MeJA solutions were supplied exogenously as root drench. It has recently been shown that the effect of JA treatment on primary metabolism, development, and defense specific traits depended on whether JA had been applied to the shoots or the roots of *Brassica oleracea* plants (Tytgat et al., 2013). We observed that root drenching with SA and SA/MeJA resulted in activation of the SA marker gene *PR1* in the leaves, whereas *VSP2* expression was activated only by MeJA treatment and not SA/MeJA treatment (**Figure 5**), indicating that application of the hormones as a root drench resulted in SA/JA crosstalk effects in the above-ground plant parts.

Treatment with MeJA led to a negative effect on leaf area and dry weight of the plants when competing with mock-treated plants (**Figure 6**). Several other studies on the costs of JA-dependent defense activation in *Arabidopsis* and in *N. attenuate* plants have provided evidence that under competition conditions the costs related to JA-dependent defenses increase (Van Dam and Baldwin, 1998; Glawe et al., 2003; Cipollini, 2007; Meldau et al., 2012). We found that activation of the SA pathway in competition-grown plants did not have major negative effects on fitness, which is similar to what Cipollini (2002) found under competition conditions, although we did detect a trend toward reduced growth by SA treatment. SA-treated plants showed higher dry weight when grown in competition with SA/MeJA-treated plants, but no effect on leaf area was found. When MeJA-treated plants competed against SA/MeJA-treated plants no significant differences in leaf area and dry weight were found, but a trend toward a reduction of the MeJA-induced fitness costs was visible by the combination with SA. Taken together, these data show that activation of the JA pathway resulted in lower fitness and lower competitive ability, while the combination of MeJA with SA did not result in an extra negative effect, but rather a trend toward a positive effect was observed. Together, this indicates that also in dense competition stands, where costs of defense activation are likely to be higher (Van Dam and Baldwin, 2001; Dietrich et al., 2005), hormonal crosstalk might be a cost-saving strategy.

However, although generally assumed, competition does not in all studied cases increase the fitness costs of defense activation. For example, in *Gossypium thurberi* and *Brassica rapa* no increase in herbivory-induced fitness costs was found in a competitive compared to a non-competitive environment (Karban, 1993; Siemens et al., 2002). Even more so, De Wit et al. (2013) showed that low red to far-red light ratios, mimicking the non-optimal light quality conditions in competitive stands, suppressed the activation of both SA- and JA-dependent defenses in *Arabidopsis*. This suggests that there could be even less fitness costs associated with defense when plants grow in competition, but unfortunately plant fitness was not investigated by De Wit et al. (2013). Together, this shows that the defense activation and fitness costs associated with plant–attacker interactions can be different depending on the competitive environment and plant species, which signifies the complexity of the mechanisms underlying these types of interactions (Karban, 1993; Van Dam and Baldwin, 1998; Siemens et al., 2002; Cipollini et al., 2003; Glawe et al., 2003; Dietrich et al., 2005; Cipollini, 2007; Meldau et al., 2012). In our experimental competition set-up it could be interesting to test (combinations of) pathogens and insects instead of hormones. This would make the results more specific for a particular plant–attacker–attacker combination and moreover, the use of living organisms instead of hormones could make the effect of induction last longer and add a disease resistance effect, which could lead to ecological costs. Furthermore, although growth rates and dry masses appear valuable phenotypic parameters to observe fitness effects, in some cases it failed to be valid predictors of effects on life time seed production (Dietrich et al., 2005). Therefore, it would be worthwhile to measure seed production in the competition set-up as well.

## Conclusion

Our results show that hormonal crosstalk during multi-attacker interactions can shift the balance between SA- or JA/ABA-dependent defenses on the one hand and JA/ET-dependent defenses on the other hand. Despite the reduced JA/ET-dependent necrotroph resistance observed during the double attacker interactions, there were no additional long-term negative fitness effects of plants that were sequentially attacked by the different attackers. Furthermore, in most cases plants grown in competition stands did not show a negative effect on plant growth in response to treatment with both SA and MeJA in comparison to the single hormone treatments. Taken together, these results suggest that hormonal crosstalk might indeed be a cost-saving strategy that allows plants to prioritize their defenses and reduce fitness costs of defense activation.

## Materials and Methods

### Plant Material and Cultivation

Seeds of *Arabidopsis thaliana* accession Col-0 were sown on river sand. Two weeks later, seedlings were transplanted into 60-ml pots containing a sand-potting soil mixture (5:12 v/v) that had been autoclaved twice for 20 min with a 24 h interval. Plants were cultivated in a growth chamber with a 10-h day and 14-h night cycle at 70% relative humidity and 21°C. Plants were watered every other day and received half-strength Hoagland solution (Hoagland and Arnon, 1938) containing 10 μM sequestreen (CIBA-Geigy, Basel, Switzerland) once a week.

### *Hyaloperonospora arabidopsidis* Inoculation

*Hyaloperonospora arabidopsidis* WACO9 was maintained on susceptible *Arabidopsis eds1* plants by weekly transfer to healthy 14-day-old seedlings as described (Koch and Slusarenko, 1990). Sporangia were obtained by washing diseased leaves in demineralized water. Debris was filtered out using Miracloth (Merck) and spores were resuspended in demineralized water to a final density of 50 spores/μl. Five-week-old plants were inoculated by spraying the *H. arabidopsidis* spore suspension using a fine paint brush, after which the plants were kept at 100% RH at 17°C for 24 h to facilitate infection (Van Damme et al., 2005).

### *Pieris rapae* Infestation

*Pieris rapae* (small cabbage white) was reared on white cabbage plants (*Brassica oleracea*) as described (Van Wees et al., 2013). First-instar caterpillars were used in all experiments. Two caterpillars were placed on fully expanded leaves of 5-week-old plants using a fine paintbrush. Caterpillars were removed 24 h later.

### *Botrytis cinerea* Inoculation

*Botrytis cinerea* inoculations were performed with strain B05.10 (Van Kan et al., 1997) as described previously (Van Wees et al., 2013). *B. cinerea* suspension with a final density of 1.10^5^ spores/ml was prepared and 5 μL droplets of the spores were applied to six leaves per plant per treatment. Plants were placed under a lid to increase relative humidity to 100% to stimulate the infection. Samples for gene expression analysis were harvested at the indicated time points. Four days after *B. cinerea* treatment, lids were removed.

### Rosette Diameter, Flowering Time, and Seed Production

Rosette diameters were measured from pictures that had been taken at the indicated time points. Two opposing longitudinal measurements were taken of each rosette using ImageJ. On-picture rulers were used to convert measured pixels to realistic centimeters. Flowering time was noted in days after treatment when the first flower appeared. To determine seed production, plants were watered every other day until they stopped producing new flowers. Inflorescences were harvested when all plants had finished flowering and the seeds were weighed on a microbalance with a 0.0001 g resolution.

### Competition Experiment

For the competition experiment, seedlings were transplanted to trays consisting of 18-ml pots organized in a 7 × 7 format, so that plants experienced competition of the aboveground plant parts, but not of the roots. Hormone treatment was applied in a chess pattern to 4-week-old plants (**Figure 4**). Only the inner nine plants were used for determining gene expression, leaf area and dry weight to circumvent an edge effect. Samples for gene expression were harvested 24 h after hormone application. Three weeks after treatment, plants were harvested and leaf area was measured using a LI-3100C Area Meter (LI-COR Environmental). Rosette dry weight was determined on a microbalance with a 0.001 g resolution when the leaves had fully dried in a 60°C stove.

### Chemical Treatments

Five-week-old plants were treated with SA (Malinkrodt Baker, Deventer, the Netherlands) or a combination of MeJA (Serva, Brunschwig Chemie, Amsterdam, the Netherlands) and ABA (Sigma, Steinheim, Germany) by dipping plants in a solution containing either 1 mM SA or a combination of 100 μM MeJA and 100 μM ABA and 0.015% (v/v) Silwet L77 (Van Meeuwen Chemicals BV, Weesp, the Netherlands). MeJA and ABA solutions were diluted from a 1000-fold concentrated stock in 96% ethanol. The mock solution contained 0.015% Silwet L77 and 0.1% ethanol.

For the competition experiment, 4-week-old plants were treated with 500 μM SA (Mallinckrodt Baker, Deventer, The Netherlands), 50 μM MeJA (Duchefa Biochemie BV, Haarlem, The Netherlands) or a combination of both by applying 3 ml of the solutions to the plants as a root drench. MeJA solution was diluted from a 1000-fold concentrated stock in 96% ethanol. The mock solution contained 0.1% ethanol.

### RNA Extraction and RT-qPCR

Total RNA was isolated as described (Oñate-Sánchez and Vicente-Carbajosa, 2008). SuperScript^TM^ III Reverse Transcriptase was used to convert DNA-free total RNA into cDNA. PCR reactions were performed in optical 384-well plates (Applied Biosystems) with an ABI PRISM^®^ 7900 HT sequence detection system using SYBR^®^ Green to monitor the synthesis of double-stranded DNA. A standard thermal profile was used: 50°C for 2 min, 95°C for 10 min, 40 cycles of 95°C for 15 s, and 60°C for 1 min. Amplicon dissociation curves were recorded after cycle 40 by heating from 60 to 95°C with a ramp speed of 1.0°C/min. Transcript levels were calculated relative to the reference gene At1g13320 (Czechowski et al., 2005) using the 2^-ΔΔCT^ method described previously (Livak and Schmittgen, 2001; Schmittgen and Livak, 2008). Primer sequences were as described (Vos et al., 2013b).

The AGI numbers of the studied genes are At2g14610 (*PR1*), At5g24770 (*VSP2*), and At5g44420 (*PDF1.2*).

## Conflict of Interest Statement

The authors declare that the research was conducted in the absence of any commercial or financial relationships that could be construed as a potential conflict of interest.

## Supplementary Material

Figure S1 **Differential expression of *PR1, VSP2*, and *PDF1.2* in response to hormone treatment and *Botrytis cinerea* infection.** RT-qPCR analysis of salicylic acid (SA)-responsive *PR1* expression **(A)**, MeJA/ABA-responsive *VSP2* expression **(B)** and *B. cinerea*-responsive *PDF1.2* expression **(A,B)**. Plants were either treated with 1 mM SA or a combination of 100 μM MeJA and 100 μM ABA. At 24 h, all plants were inoculated with *B. cinerea*. Samples were taken at the indicated time points after the first treatment. Different letters indicate a statistically significant difference between the different treatments within one time point (ANOVA, Tukey *post hoc* test; *P* < 0.05; NS, not significant). Error bars represent SE, *n* = 3 plants.

Figure S2 **Effect of hormone application on disease resistance against *B. cinerea*.** Quantification of disease symptoms of *Arabidopsis* Col-0 plants infected with *B. cinerea*. Twenty-four hour before inoculation with *B. cinerea*, plants were treated with 1 mM SA or a combination of 100 μM MeJA and 100 μM ABA. Disease severity of the inoculated leaves was scored in three classes. Percentage of leaves in each class was calculated per plant (*X*^2^-test; *n* = 20 plants).

Figure S3 **Growth and fitness parameters of single- and double-treated plants.** Rosette diameter (cm), flowering time (days post inoculation), and total seed production (mg) of *Arabidopsis* plants. Plants were either treated with 1 mM SA **(A)** or a combination of 100 μM MeJA and 100 μM ABA **(B)**. At 24 h all plants were inoculated with *B. cinerea* **(A,B)**. Different letters indicate a statistically significant difference between the different treatments (ANOVA, Tukey *post hoc* test; *P* < 0.05). Error bars represent SE, *n* = 20 plants.
