# Supplementary figures and images for: Impact of hormonal crosstalk on plant resistance and fitness under multi-attacker conditions
Source: Front Plant Sci. 2015 Aug 17;6:639. doi: 10.3389/fpls.2015.00639 (PMC4538242; doi:10.3389/fpls.2015.00639)

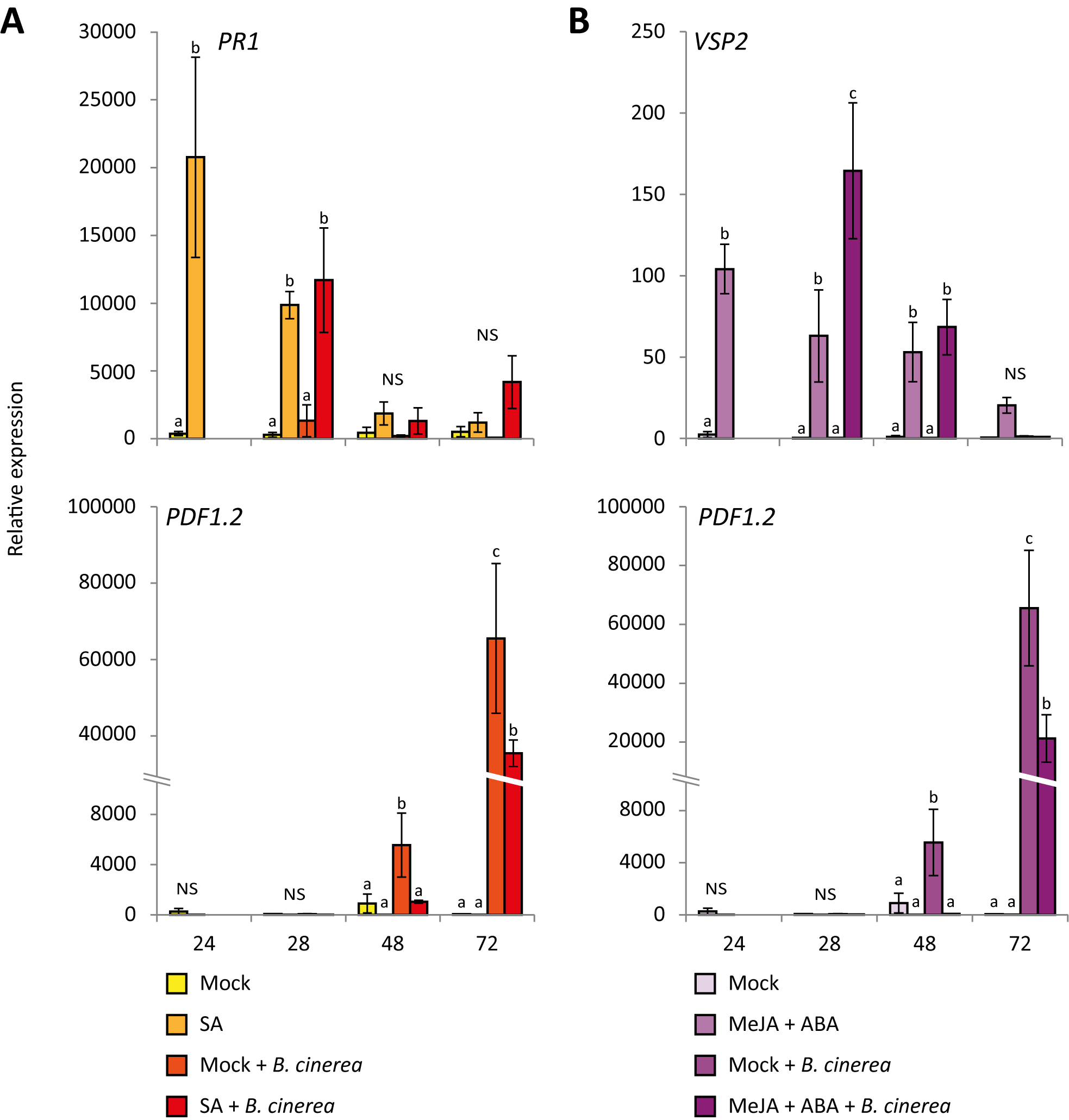

Supplement: Figure S1 — Differential expression of PR1, VSP2, and PDF1.2 in response to hormone treatment and Botrytis cinerea infection. RT-qPCR analysis of salicylic acid (SA)-responsive PR1 expression (A), MeJA/ABA-responsive VSP2 expression (B) and B. cinerea-responsive PDF1.2 expression (A,B). Plants were either treated with 1 mM SA or a combination of 100 μM MeJA and 100 μM ABA. At 24 h, all plants were inoculated with B. cinerea. Samples were taken at the indicated time points after the first treatment. Different letters indicate a statistically significant difference between the different treatments within one time point (ANOVA, Tukey post hoc test; P < 0.05; NS, not significant). Error bars represent SE, n = 3 plants. [file Supplemental_Figure1.TIF]

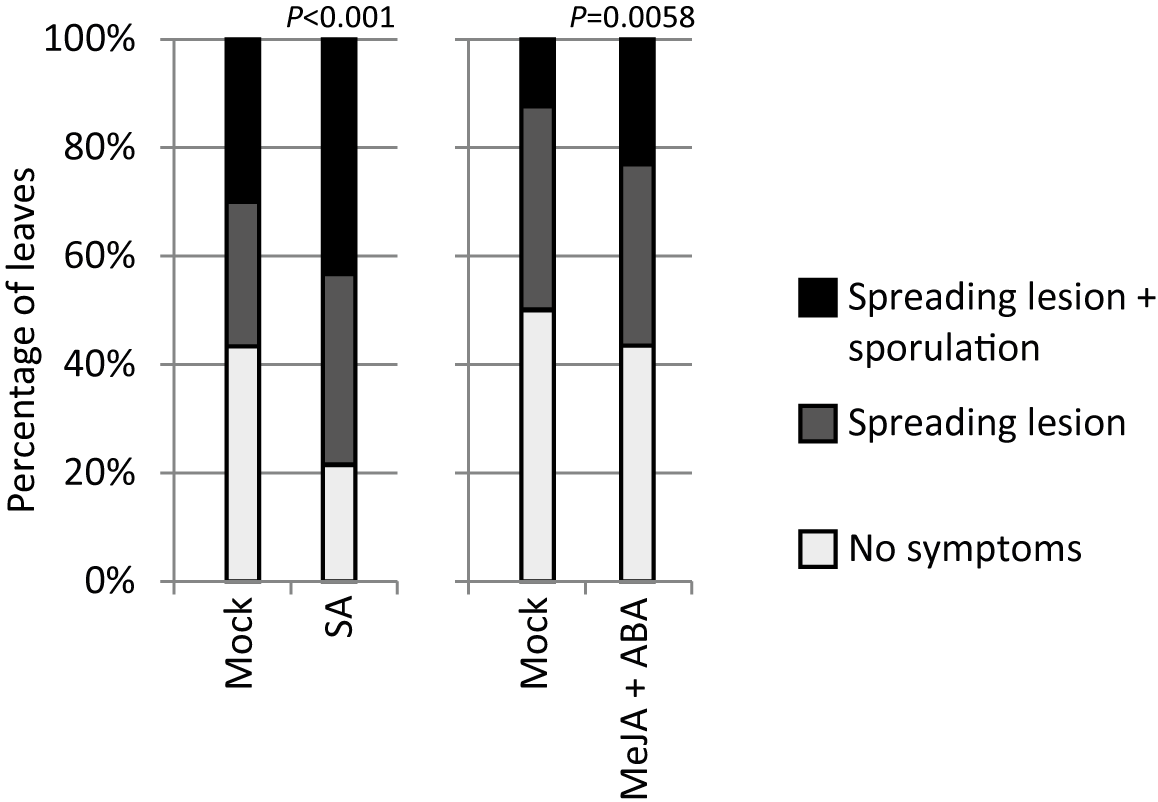

Supplement: Figure S2 — Effect of hormone application on disease resistance against B. cinerea. Quantification of disease symptoms of Arabidopsis Col-0 plants infected with B. cinerea. Twenty-four hour before inoculation with B. cinerea, plants were treated with 1 mM SA or a combination of 100 μM MeJA and 100 μM ABA. Disease severity of the inoculated leaves was scored in three classes. Percentage of leaves in each class was calculated per plant (X2-test; n = 20 plants). [file Supplemental_Figure2.TIF]

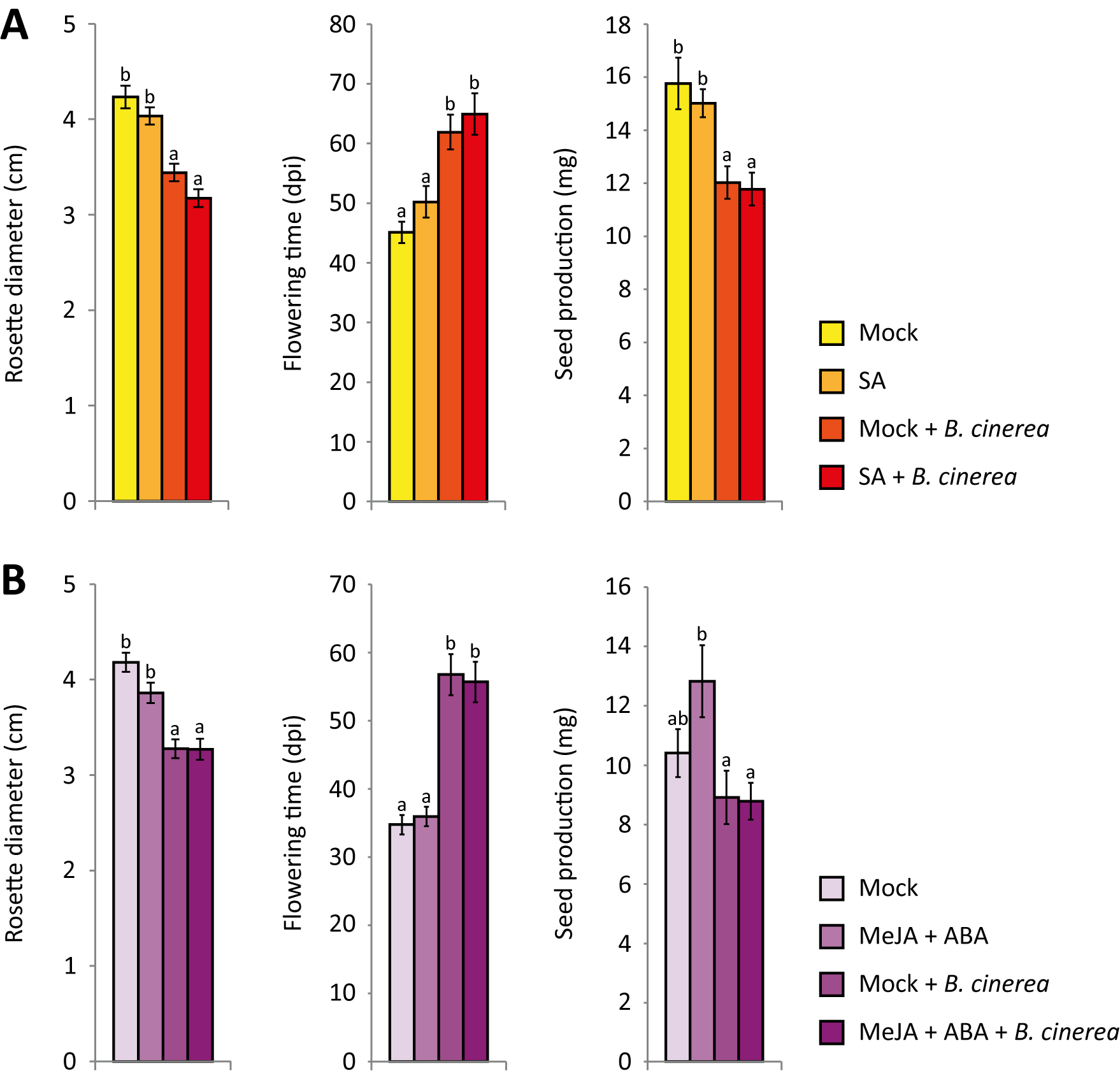

Supplement: Figure S3 — Growth and fitness parameters of single- and double-treated plants. Rosette diameter (cm), flowering time (days post inoculation), and total seed production (mg) of Arabidopsis plants. Plants were either treated with 1 mM SA (A) or a combination of 100 μM MeJA and 100 μM ABA (B). At 24 h all plants were inoculated with B. cinerea (A,B). Different letters indicate a statistically significant difference between the different treatments (ANOVA, Tukey post hoc test; P < 0.05). Error bars represent SE, n = 20 plants. [file Supplemental_Figure3.TIF]
